# Regulation of ATP-Dependent Proteolysis by Membrane-Anchored Assemblies

**DOI:** 10.1063/4.0000871

**Published:** 2025-10-27

**Authors:** Alireza Ghanbarpour, Naseer Iqbal

**Affiliations:** 1 Washington University School of Medicine, St. Louis,, MO, USA; 2 Washington University School of Medicine, St. Louis, MO, USA

## Abstract

Protein degradation by AAA+ proteases is essential for bacterial adaptation to environmental stress. The membrane-bound AAA+ protease FtsH plays a central role in this process by degrading both membrane- associated and soluble substrates across diverse organisms. FtsH functions as a homohexamer composed of genetically linked AAA+ and zinc metallopeptidase domains, along with membrane-spanning and periplasmic regions. Following recognition of the degron sequence by the AAA+ module, substrate proteins are unfolded through ATP hydrolysis and subsequently translocated into the protease chamber for degradation.

In *Escherichia coli*, FtsH functions within a ∼1.8 MDa inner membrane complex formed in association with the SPFH (Stomatin, Prohibitin, Flotillin, HflK/C) family proteins HflK and HflC. This FtsH•HflK/C assembly is essential for bacterial survival under aminoglycoside-induced proteotoxic stress, though the underlying mechanism has remained unclear. Recent cryo-EM studies revealed that the HflK/C assembly can adopt both closed, symmetric and open, asymmetric conformations, raising important questions about which conformation represents the biologically active state and how these structural states regulate FtsH proteolysis.

To address this, we engineered a disulfide-stabilized HflK/C variant locked in the closed conformation and validated its structure using high- resolution cryo-EM. Phenotypic assays showed that cells expressing either this locked variant or a mutant that disrupts the FtsH–HflK/C interaction exhibit impaired growth under aminoglycoside stress, highlighting the functional importance of HflK/C dynamics. Surprisingly, cryo-EM analysis of the FtsH•HflK/C complex isolated from aminoglycoside-exposed cells revealed a novel HflK/C architecture with two opposing openings, suggesting a structural remodeling that may facilitate membrane substrate recruitment. Together, our results demonstrate that both the dynamic architecture of the HflK/C assembly and its specific interaction with FtsH are essential for proteolytic adaptation to proteotoxic stress. Given the conserved structural and functional features of SPFH-domain proteins, our study may provide a foundation for understanding the molecular functions of these massive membrane assemblies across both prokaryotic and eukaryotic systems.

